# Efficacy and safety of mechanical thrombectomy in distal medium middle cerebral artery occlusion ischemic stroke patients on low-dose aspirin

**DOI:** 10.1177/17474930251317883

**Published:** 2025-01-28

**Authors:** Hamza Adel Salim, Vivek Yedavalli, Fathi Milhem, Basel Musmar, Nimer Adeeb, Motaz Daraghma, Kareem El Naamani, Nils Henninger, Sri Hari Sundararajan, Anna Luisa Kühn, Jane Khalife, Sherief Ghozy, Luca Scarcia, Benjamin YQ Tan, Robert W Regenhardt, Jeremy J Heit, Nicole M Cancelliere, Joshua D Bernstock, Aymeric Rouchaud, Jens Fiehler, Sunil Sheth, Ajit S Puri, Christian Dyzmann, Marco Colasurdo, Leonardo Renieri, João Pedro Filipe, Pablo Harker, Răzvan Alexandru Radu, Mohamad Abdalkader, Piers Klein, Thomas R Marotta, Julian Spears, Takahiro Ota, Ashkan Mowla, Pascal Jabbour, Arundhati Biswas, Frédéric Clarençon, James E Siegler, Thanh N Nguyen, Ricardo Varela, Amanda Baker, Muhammed Amir Essibayi, David Altschul, Nestor R Gonzalez, Markus A Möhlenbruch, Vincent Costalat, Benjamin Gory, Christian Paul Stracke, Constantin Hecker, Hamza Shaikh, Christoph J Griessenauer, David S Liebeskind, Alessandro Pedicelli, Andrea M Alexandre, Illario Tancredi, Tobias D Faizy, Erwah Kalsoum, Max Wintermark, Boris Lubicz, Aman B Patel, Vitor Mendes Pereira, Adrien Guenego, Adam A Dmytriw

**Affiliations:** 1 Division of Neuroradiology, Department of Radiology, Johns Hopkins Medical Center, Baltimore, MD, USA; 2 Neuroendovascular Program, Massachusetts General Hospital and Brigham and Women’s Hospital, Harvard Medical School, Harvard University, Boston, MA, USA; 3 Department of Neurosurgery, Thomas Jefferson University, Philadelphia, PA, USA; 4 Department of Neurosurgery and Interventional Neuroradiology, Louisiana State University, Baton Rouge, LA, USA; 5 Department of Neurology, University of Massachusetts Chan Medical School, Worcester, MA, USA; 6 Department of Endovascular Neurosurgery and Neuroradiology NJMS, Newark, NJ, USA; 7 Division of Neurointerventional Radiology, Department of Radiology, University of Massachusetts Medical Center, Worcester, MA, USA; 8 Cooper Neurological Institute, Cooper University Hospital, Cooper Medical School of Rowan University, Camden, NJ, USA; 9 Departments of Neurological Surgery & Radiology, Mayo Clinic, Rochester, MN, USA; 10 Department of Neuroradiology, Henri Mondor Hospital, Creteil, France; 11 Department of Medicine, Yong Loo Lin School of Medicine, National University of Singapore, Singapore; 12 Division of Neurology, Department of Medicine, National University Hospital, Singapore; 13 Department of Interventional Neuroradiology, Stanford Medical Center, Palo Alto, CA, USA; 14 Neurovascular Centre, Divisions of Therapeutic Neuroradiology and Neurosurgery, St. Michael’s Hospital, University of Toronto, Toronto, ON, Canada; 15 Department of Neurosurgery, Brigham and Women’s Hospital, Harvard Medical School, Boston, MA, USA; 16 University Hospital of Limoges, Neuroradiology Department, Dupuytren, Université de Limoges, XLIM CNRS, UMR 7252, Limoges, France; 17 Department of Diagnostic and Interventional Neuroradiology, University Medical Center Hamburg-Eppendorf, Hamburg, Germany; 18 Department of Neurology, UTHealth McGovern Medical School, Houston, TX, USA; 19 Neuroradiology Department, Sana Kliniken, Lübeck GmbH, Lübeck, Germany; 20 Department of Interventional Radiology, Oregon Health and Science University, Portland, OR, USA; 21 Interventistica Neurovascolare, Ospedale Careggi di Firenze, Florence, Italy; 22 Department of Diagnostic and Interventional Neuroradiology, Centro Hospitalar Universitário do Porto, Porto, Portugal; 23 Department of Neurology, University of Cincinnati Medical Center, Cincinnati, OH, USA; 24 Department of Neuroradiology, Gui de Chauliac Hospital, Montpellier University Medical Center, Montpellier, France; 25 Departments of Radiology & Neurology, Boston Medical Center, Boston, MA, USA; 26 Department of Neurosurgery, Tokyo Metropolitan Tama Medical Center, Tokyo, Japan; 27 Division of Stroke and Endovascular Neurosurgery, Department of Neurological Surgery, Keck School of Medicine, University of Southern California (USC), Los Angeles, CA, USA; 28 Department of Neurosurgery, Westchester Medical Center at New York Medical College, Valhalla, NY, USA; 29 Department of Neuroradiology, Pitié-Salpêtrière Hospital, Paris, France; 30 GRC BioFast, Sorbonne University, Paris VI, Paris, France; 31 Department of Neurology, Centro Hospitalar Universitário do Porto, Porto, Portugal; 32 Department of Neurological Surgery and Montefiore-Einstein Cerebrovascular Research Lab, Montefiore Medical Center, Albert Einstein College of Medicine, Bronx, NY, USA; 33 Department of Neurosurgery, Cedars-Sinai Medical Center, Los Angeles, CA, USA; 34 Sektion Vaskuläre und Interventionelle Neuroradiologie, Universitätsklinikum Heidelberg, Heidelberg, Germany; 35 Department of Interventional Neuroradiology, Nancy University Hospital, Nancy, France; 36 INSERM U1254, IADI, Université de Lorraine, Nancy, France; 37 Department of Radiology, Interventional Neuroradiology Section, University Medical Center Münster, Münster, Germany; 38 Departments of Neurology & Neurosurgery, Christian Doppler Clinic, Paracelsus Medical University Salzburg, Salzburg, Austria; 39 UCLA Stroke Center and Department of Neurology Department, UCLA, Los Angeles, CA, USA; 40 UOSA Neuroradiologia Interventistica, Fondazione Policlinico Universitario A. Gemelli IRCCS, Roma, Italy; 41 Department of Neurology, Hôpital Civil Marie Curie, Charleroi, Belgium; 42 Department of Radiology, Neuroendovascular Program, University Medical Center Münster, Münster, Germany; 43 Department of Neuroradiology, MD Anderson Medical Center, Houston, TX, USA; 44 Department of Diagnostic and Interventional Neuroradiology, Erasme University Hospital, Brussels, Belgium

**Keywords:** Acute ischemic stroke, distal medium vessel occlusions, mechanical thrombectomy, aspirin

## Abstract

**Background:**

Acute ischemic stroke (AIS) from distal medium vessel occlusion (DMVO) presents unique treatment challenges. Mechanical thrombectomy (MT) is emerging as a viable option for these patients, yet the role of pre-stroke aspirin treatment is unclear. This study evaluates the impact of pre-stroke low-dose aspirin on outcomes in DMVO patients undergoing MT.

**Methods:**

We conducted a multinational, multicenter, propensity score-weighted analysis within the Multicenter Analysis of primary Distal medium vessel occlusions: effect of Mechanical Thrombectomy (MAD-MT) registry. Patients with AIS due to DMVO, treated with MT, were included. We compared outcomes between patients on pre-stroke low-dose aspirin (75–100 mg) and those not on antiplatelet therapy. The primary outcome was functional independence at 90 days (modified Rankin Scale (mRS), 0–2). Secondary outcomes included excellent functional outcome at 90 days (mRS, 0–1), mortality, and day 1 post-MT National Institutes of Health Stroke Scale (NIHSS) score. Safety outcomes focused on hemorrhagic complications, including symptomatic intracerebral hemorrhage (sICH).

**Results:**

Among 1354 patients, 150 were on pre-stroke low-dose aspirin. After applying inverse probability of treatment weighting (IPTW), aspirin use was associated with significantly better functional outcomes (mRS, 0–2: odds ratio (OR) = 1.89, 95% confidence interval (CI) = 1.14 to 3.12) and lower 90-day mortality (OR = 0.56, 95% CI = 0.32 to 1.00). The aspirin group had lower NIHSS scores on day 1 (β = −1.5, 95% CI = −2.8 to −0.27). The sICH rate was not significantly different between the groups (OR = 0.92, 95% CI = 0.60 to 1.43).

**Conclusions:**

Pre-stroke low-dose aspirin was associated with improved functional outcomes and reduced mortality in patients with DMVO undergoing MT, without a significant increase in sICH. These findings suggest that low-dose aspirin may be safe and associated with more frequent excellent outcomes for this patient population. Further prospective studies are needed to validate these results and assess long-term outcomes.

## Introduction

Acute ischemic stroke (AIS) is a significant contributor to global disability and mortality. A notable portion of AIS cases is due to distal medium vessel occlusion (DMVO), estimated to be 25–40%.^1–14^ Traditionally, intravenous thrombolysis (IVT) has been the primary treatment for AIS caused by DMVO.^15,16^ However, advancements in endovascular techniques have led to an increased focus on mechanical thrombectomy (MT) as an alternative treatment option. This shift is supported by several observational studies and ongoing randomized controlled trials (RCTs) investigating the potential benefits of MT for select DMVO patients.^15,17–30^

Aspirin, a widely used antiplatelet medication, has shown effectiveness in preventing thrombotic events during neurointerventional procedures.^31–33^ Given that thromboembolic events are a primary mechanism of AIS, the impact of aspirin during MT in patients with DMVO remains unclear. While aspirin’s antiplatelet and anti-inflammatory properties may reduce these risks and provide neuroprotective benefits, these potential beneficial effects may be offset by an increased risk of hemorrhagic complications. Thus, a careful evaluation of the presumed safety and efficacy of MT in DMVO patients treated with aspirin is necessary^34–36^

Although many antiplatelet agents are used in cardiovascular disease prevention, whether as primary, secondary, or tertiary stage, low-dose aspirin remains the most widely used, particularly for primary prevention.^37,38^

Data regarding the efficacy and safety of MT in patients who were taking aspirin before stroke onset are scarce.^39^ While studies have reported the feasibility of MT in patients on aspirin with large vessel occlusion stroke,^40–46^ none has evaluated this in DMVO. In this study, we aim to evaluate the efficacy and safety of pre-stroke low-dose aspirin in DMVO stroke patients undergoing MT.

## Methods

Our study is an analysis performed within the Multicenter Analysis of primary Distal medium vessel occlusions: effect of Mechanical Thrombectomy (MAD-MT) registry.^17,36,47–59^ This article adheres to the Strengthening the Reporting of Observational Studies in Epidemiology (STROBE) guidelines.^60,61^

### Ethical considerations and study approval

The institutional review board or local ethical standards committee at each of the 37 participating sites across North America, Asia, and Europe granted approval for this study. Due to the study’s retrospective nature, informed consent was waived. All data from this study’s findings are available from the corresponding author upon reasonable request.

### Study population and inclusion criteria

We focused on patients with AIS due to DMVO as previously described.^17,47,54,55^ Inclusion criteria were as follows: (1) AIS patients with DMVO in the M2, M3, and M4 segments of the middle cerebral artery (MCA) defined according to Saver et al.’s^15^ criteria and (2) those undergoing MT with or without IVT. Exclusion criteria were as follows: (1) patients with incomplete antiplatelet data, taking antiplatelet agents other than aspirin (e.g. clopidogrel, cilostazol), or taking aspirin at a dosage higher than 100 mg; (2) patients with M2 segments classified as dominant or large vessel occlusion (LVO); and (3) patients treated exclusively with intra-arterial thrombolytic agents.

### Data collection process

Data collection involved consecutive patients from September 2017 to July 2023. Local neurointerventionalists or vascular neurologists assessed angiographic treatment success before forwarding data to the MAD-MT consortium. Each center self-reported these data.

We recorded comprehensive baseline clinical and demographic characteristics, including sex, age, hypertension, hypercholesterolemia, diabetes mellitus, atrial fibrillation, and smoking status. Furthermore, we documented the pre-stroke modified Rankin Scale (mRS) score, the occluded vessel, and National Institutes of Health Stroke Scale (NIHSS) score at presentation. In addition, patients were stratified into subgroups based on their occlusion location during the initial angiography, differentiating between medium (M2) and distal branches (M3, M4) of the MCA.

Additional data of interest encompassed antiplatelet and anticoagulation medication status. Patients were classified as being on antiplatelet therapy if they were taking any home antiplatelet medications (e.g. aspirin, clopidogrel, prasugrel, cilostazol) upon presentation. Similarly, patients were considered anticoagulated if they were on home vitamin K antagonists or direct oral anticoagulants at the time of presentation. Moreover, time from symptom onset to arterial puncture and recanalization, anesthesia type, access site (femoral or radial), and post-MT imaging modalities (computed tomography (CT), magnetic resonance (MR), or none) were recorded.

### Procedural and technical details

Treatment consisted of MT alone or IVT + MT. MT access site was at the individual operator’s discretion. Similarly, the technique (aspiration, stent retriever, or combination) and number of passes were left to the treating physician’s discretion.

For patients in the combined MT + IVT group, IVT was given as per institutional protocol, using either alteplase or tenecteplase as per standard clinical guidelines. Alteplase was given at a standard dose of 0.9 mg/kg, with 10% of the total dose delivered as an initial bolus followed by an infusion of the remaining 90% over 60 min. Tenecteplase was administered in a single intravenous bolus at a dose of 0.25 mg/kg, infused over 5–10 s.

### Outcomes

The primary outcome was functional independence at 90 days (mRS, 0–2), with secondary outcomes including excellent outcome at 90 days (mRS, 0–1), mortality (mRS, 6), and day 1 post-MT NIHSS score. Safety outcomes were hemorrhagic complications of any type and symptomatic intracerebral hemorrhage (sICH), defined according to “The Heidelberg Bleeding Classification.”^62^

### Statistical analysis

We employed inverse probability of treatment weighting (IPTW) to balance the distribution of confounding variables between the MT and medical management (MM) groups. The selection of propensity score (PS) weights over a regression-based, covariate-adjustment technique such as logistic regression was influenced by several considerations. To minimize residual confounding in observational studies,^63^ a large number of variables can be summarized using PS techniques. Furthermore, notable variations were seen in the distribution of specific baseline features among the two groups. In addition, propensity score weights serve to mitigate the potential for bias by estimating scores in a manner that is independent of the outcome.^64^

The estimation of PS weights was conducted using generalized boosted modeling (GBM) methodology. GBM is a machine learning multivariate non-parametric regression technique that estimates the PS of individuals iteratively to maximize balance in observed covariables.^65,66^ GBM has the capability to integrate interactions among numerous factors, hence mitigating the potential for model misspecification.^66,67^ Prior studies have demonstrated that GBM provides superior performance compared to logistic regression in PS estimation.^68^ The current analyses utilized GBM as a method to mitigate the potential bias in results that may arise due to model misspecification.^65^ The model included sex, age, hypercholesterolemia, occlusion location, hypertension, diabetes mellitus, atrial fibrillation presence, IVT, baseline NIHSS scores, pre-stroke mRS scores, pre-stroke use of anticoagulants, Alberta Stroke Program Early CT Score (ASPECTS), and stroke onset to puncture time, with the aim of estimating the average treatment effect (ATE). The mean Kolmogorov–Smirnov (KS.mean) statistic was employed as a stopping criterion for assessing and summarizing balance across pretreatment variables. A 10,000-tree GBM model with an interaction depth of 3, a shrinkage value of 0.01, and a bag fraction of 1 was utilized.

The analyses used balance diagnostics for PS weights, as per known practices.^69^ The diagnostic analysis primarily examined the absolute standardized mean difference (ASMD) between weighted and unweighted variables. In our analysis, we deemed any ASMD greater than 0.10 to indicate covariate imbalance. The literature has proposed that any value equal to or below 0.10 can be considered as a trivial difference in terms of relative balance.^70,71^ To minimize the mean square error a doubly robust estimation was employed, wherein additional adjustments were made for the covariates using weighted multivariable logistic regression analysis.^65,72^ All statistical analyses were performed using R Studio Version 4.2.2. No imputations were made, and adjustments for multiple testing were not performed.

## Results

### Patient demographics and baseline characteristics

A total of 1354 patients were included in the study (median age 74 years; interquartile range (IQR), 62–82, 638 males, 715 females), with 1204 patients not on antiplatelet therapy and 150 patients on low-dose aspirin. Table 1 provides a summary of baseline characteristics.

**Table 1.** Baseline characteristics.

Variable	Overall	No antiplatelet	Aspirin (75–100 mg)	p^a^
	N = 1354	N = 1204	N = 150	
Male, n (%)	638 (47)	562 (47)	76 (51)	0.36
Age, median (IQR)	74 (62, 82)	74 (62, 82)	75 (68, 82)	0.1
Hypercholesterolemia, n (%)	399 (30)	316 (27)	83 (55)	<0.001
Hypertension, n (%)	875 (65)	773 (64)	102 (68)	0.37
Site of initial occlusion, n (%)				0.26
Medium (M2)	1177 (87)	1051 (87)	126 (84)	
Distal (M3, M4)	177 (13)	153 (13)	24 (16)	
Diabetes, n (%)	279 (21)	241 (20)	38 (25)	0.13
Atrial fibrillation, n (%)	492 (36)	432 (36)	60 (40)	0.33
Current smokers, n (%)	193 (14)	157 (13)	36 (24)	<0.001
Previous use of antiplatelet drugs, n (%)	150 (11)	0 (0)	150 (100)	<0.001
Previous use of anticoagulant drugs, n (%)	342 (27)	315 (28)	27 (18)	0.012
Pre-stroke mRS, n (%)				0.6
0	861 (67)	753 (66)	108 (72)	
1	161 (13)	145 (13)	16 (11)	
2	109 (8.5)	100 (8.8)	9 (6.0)	
3	108 (8.4)	95 (8.4)	13 (8.7)	
4	46 (3.6)	42 (3.7)	4 (2.7)	
ASPECTS, median (IQR)	9.00 (8.00, 10.00)	9.00 (8.00, 10.00)	8.00 (7.00, 10.00)	0.002
Baseline NIHSS, median (IQR)	10 (6, 17)	10 (6, 17)	9 (5, 17)	0.31

mRS: modified Rankin Scale; NIHSS: National Institutes of Health Stroke Scale; ASPECTS: Alberta Stroke Program Early CT Score.

a Pearson’s chi-square test; Wilcoxon rank-sum test; Fisher’s exact test.

Notable differences were observed in the prevalence of hypercholesterolemia (27% in no antiplatelet vs 55% in aspirin group, p < 0.001) and current smokers (13% in no antiplatelet vs 24% in aspirin group, p < 0.001). Previous use of anticoagulant drugs was higher in the no antiplatelet group (28% vs 18%, p = 0.012). The median ASPECTS was slightly lower in the aspirin group (8.00, IQR, 7.00–10.00) compared to the no antiplatelet group (9.00, IQR, 8.00–10.00, p = 0.002). Other baseline characteristics, including hypertension, diabetes, atrial fibrillation, and baseline NIHSS scores, showed no significant differences between the groups. Balance diagnostics for various patient characteristics after propensity score weighting are presented in Supplementary Table 1.

### Periprocedural details

Supplementary Table 2 presents the periprocedural details. The administration of IVT was comparable between the groups (45% in the aspirin group vs 47% in the no antiplatelet group; p = 0.78). A significant difference was observed in the first-line technique used, with a higher proportion of aspiration techniques in the aspirin group (24% vs 18% in the no antiplatelet group; p = 0.002).

The median onset to arterial puncture time was shorter in the aspirin group (230 min, IQR, 154–379) compared to the no antiplatelet group (270 min, IQR, 180–442; p = 0.007). Similarly, the median onset to recanalization time was shorter in the aspirin group (294 min, IQR, 193–440) compared to the no antiplatelet group (330 min, IQR, 231–514; p = 0.006). The use of general anesthesia was more common in the aspirin group (58% vs 26%; p < 0.001).

### Outcomes

Supplementary Table 3 shows the outcomes of the univariable crude analysis and the doubly robust IPTW evaluation. The 90-day mRS 0–2 was better in the aspirin group (mRS 0–2: IPTW model odds ratio (OR) = 1.89, 95% confidence interval (CI), 1.14 to 3.12, p = 0.013). Although the proportion of patients achieving mRS 0–1 was higher in the aspirin group, the difference between the two groups was not statistically significant (mRS 0–1: IPTW model OR = 1.62, 95% CI, 0.98 to 2.67, p = 0.06). Patients in the aspirin group had a significantly lower NIHSS score on day 1 (median 4, IQR, 2–12) compared to the no antiplatelet group (median 6, IQR, 2–14, crude analysis: β = −2.2, [95% CI, −3.9 to −0.55], p = 0.009; IPTW model: β = −1.5, [95% CI, −2.8 to −0.27], p = 0.018). However, there were no significant differences in the rates of thrombolysis in cerebral infarction (TICI) 2b-3 and TICI 2c-3 between the groups.

Mortality at 90 days was lower in the aspirin group (13% vs 17% in no antiplatelet, IPTW model OR = 0.56, 95% CI, 0.32 to 1.00, p = 0.048). The aspirin group also had a lower incidence of HI1 type ICH (IPTW model OR = 0.12, 95% CI, 0.05 to 0.31, p < 0.001) and a higher incidence of PH2 type ICH (IPTW model OR = 5.80, 95% CI, 2.43 to 13.8, p < 0.001). The rates of HI2, PH1, and subarachnoid hemorrhage (SAH) were not statistically significant between the comparable groups.

## Discussion

In this multicenter multinational study, we examined a total of 1354 MCA DMVO stroke patients, comparing MT outcomes between those on low-dose aspirin and no antiplatelet therapy. Pre-stroke low-dose aspirin use was significantly associated with higher functional independence (mRS, 0–2), lower day 1 post-MT NIHSS scores at follow-up, and a reduced mortality rate at 90 days without significant increase in the rate of sICH.

Aspirin use in the primary prevention of cardiovascular disease has been shown to decrease all-cause mortality, including major cardiovascular complications such as myocardial infarction and ischemic stroke. However, it simultaneously increases the risk of hemorrhagic stroke and bleeding.^38,73,74^ Daily aspirin use (160–300 mg) started within the first 48 h of ischemic stroke symptom onset is associated with reduced morbidity and mortality.^75^

Aspirin enhances circulation by preventing platelet aggregation, the initial step in thrombus formation, through the irreversible inhibition of COX-1 and COX-2 via acetylation.^76^ This is particularly relevant in DMVO strokes, where many studies suggest that thrombi in distal occlusions have different compositions, being RBC-rich, fresher, less compact, and more responsive to treatment,^77^ in contrast to the fibrin-rich thrombi of atherosclerotic plaque origin, which are mostly responsible for more proximal occlusions. In addition, the structural differences between large and medium-sized arteries play a role. Large arteries have a thicker tunica media and a slightly thicker tunica adventitia.^78^ The tunica media, composed of smooth muscles controlled by the sympathetic and parasympathetic nervous systems, contracts in response to ischemic stroke, leading to vasoconstriction and reducing the effectiveness of antiplatelet therapy in preventing artery occlusion by thrombus compared to medium vessels.^62,79^ Nonetheless, further research is necessary to confirm this phenomenon.

Many studies on the safety of using aspirin during IVT have demonstrated an increased risk of bleeding and mortality.^75^ However, a recent retrospective study conducted in the United Kingdom showed contradictory results, suggesting that intravenous aspirin during emergent MT is associated with good clinical outcomes and does not increase the risk of ICH, even when combined with IVT.^80^ Our results align with a Chinese observational study that showed better clinical outcomes in patients taking single or dual antiplatelet therapy than no antiplatelet therapy.^81^ However, contradictory results were observed in a nationwide prospective study, which showed no improvement in clinical outcomes and functional independence with antiplatelet monotherapy before MT.^39^ The improved functional outcomes in our cohort’s low-dose aspirin group may be due to the type of vessels studied. While the nationwide study focused on LVOs, our research was confined to DMVOs.

Furthermore, our results contradicted another study, MR CLEAN, which found no evidence of better functional outcomes in patients with baseline antiplatelet therapy before MT for LVO.^82^ This can be explained by the fact that the previous studies were conducted on patients with baseline antiplatelet therapy in general, regardless of the type, and did not focus on the specific use of any one type.^39,82^ However, our study focused only on using low-dose aspirin as the baseline antiplatelet and on MT for DMVO.

Our findings indicate that while the rate of some ICH subtypes in the aspirin group were higher, most hemorrhages were asymptomatic. This observation is supported by the lack of significant difference in the occurrence of sICH between the two cohorts. Although patients on aspirin had a lower incidence of HI type 1 ICH, they had a higher incidence of PH2 type ICH. These results are conflict with the MR CLEAN study, which showed a significant increase in the risk of ICH in patients on baseline antiplatelet therapy after MT.^82^ This discrepancy may be explained by the fact that the no antiplatelet group had a slightly higher percentage of previous anticoagulant use, which may increase the risk of sICH. However, our results are consistent with the findings of the nationwide study,^39^ the Chinese study,^81^ and the two smaller studies,^82,83^ where no significant increase in ICH risk was observed.

Our findings suggest that MT is safe in patients taking pre-stroke aspirin, with improved outcomes among patients taking aspirin seeming to be more pronounced in DMVO compared to LVO. However, further validation via prospective studies is needed.

Our study has multiple strengths, including large-scale, multinational, multicenter, and real-world data, thereby improving generalizability. However, our study is not without limitations. The primary limitation is that the indication for baseline aspirin use was not recorded. Nonetheless, the exact reason for aspirin use may have a limited impact on our findings as our focus was on the safety of performing MT in patients already on aspirin, irrespective of its initial indication, rather than on the indication for prescribing aspirin before MT. Future studies should consider recording the baseline indication to assess its potential contribution to outcomes.

Other limitations include, first, the retrospective nature of the analysis introduces the possibility of selection bias, potentially influencing the generalizability of the findings. Second, the scope of the study is limited to a 90-day post-intervention follow-up period, which restricts our insight into the long-term repercussions of MT complications on patients’ functional recovery and overall quality of life. Third, we did not include patients who had higher aspirin dose prior to MT, so we do not know if our results are generalizable to this subgroup of patients. Finally, the exact cause of stroke was not documented, which limits our ability to draw conclusions about the underlying etiology.

## Conclusion

In conclusion, our findings suggest that MT for DMVOs is safe for patients with pre-stroke low-dose aspirin use. This association with improved functional outcomes and reduced mortality, without a significant increase in sICH, highlights aspirin’s potential role in enhancing treatment efficacy. However, caution is warranted, as pre-stroke aspirin is linked to a higher incidence of PH2-type ICH. Further prospective studies are needed to validate these results and explore the long-term effects of aspirin use in this patient population.

## Supplemental Material

sj-docx-1-wso-10.1177_17474930251317883 – Supplemental material for Efficacy and safety of mechanical thrombectomy in distal medium middle cerebral artery occlusion ischemic stroke patients on low-dose aspirin Supplemental material, sj-docx-1-wso-10.1177_17474930251317883 for Efficacy and safety of mechanical thrombectomy in distal medium middle cerebral artery occlusion ischemic stroke patients on low-dose aspirin by Hamza Adel Salim, Vivek Yedavalli, Fathi Milhem, Basel Musmar, Nimer Adeeb, Motaz Daraghma, Kareem El Naamani, Nils Henninger, Sri Hari Sundararajan, Anna Luisa Kühn, Jane Khalife, Sherief Ghozy, Luca Scarcia, Benjamin YQ Tan, Robert W Regenhardt, Jeremy J Heit, Nicole M Cancelliere, Joshua D Bernstock, Aymeric Rouchaud, Jens Fiehler, Sunil Sheth, Ajit S Puri, Christian Dyzmann, Marco Colasurdo, Leonardo Renieri, João Pedro Filipe, Pablo Harker, Răzvan Alexandru Radu, Mohamad Abdalkader, Piers Klein, Thomas R Marotta, Julian Spears, Takahiro Ota, Ashkan Mowla, Pascal Jabbour, Arundhati Biswas, Frédéric Clarençon, James E Siegler, Thanh N Nguyen, Ricardo Varela, Amanda Baker, Muhammed Amir Essibayi, David Altschul, Nestor R Gonzalez, Markus A Möhlenbruch, Vincent Costalat, Benjamin Gory, Christian Paul Stracke, Constantin Hecker, Hamza Shaikh, Christoph J Griessenauer, David S Liebeskind, Alessandro Pedicelli, Andrea M Alexandre, Illario Tancredi, Tobias D Faizy, Erwah Kalsoum, Max Wintermark, Boris Lubicz, Aman B Patel, Vitor Mendes Pereira, Adrien Guenego and Adam A Dmytriw in International Journal of Stroke

sj-docx-2-wso-10.1177_17474930251317883 – Supplemental material for Efficacy and safety of mechanical thrombectomy in distal medium middle cerebral artery occlusion ischemic stroke patients on low-dose aspirin Supplemental material, sj-docx-2-wso-10.1177_17474930251317883 for Efficacy and safety of mechanical thrombectomy in distal medium middle cerebral artery occlusion ischemic stroke patients on low-dose aspirin by Hamza Adel Salim, Vivek Yedavalli, Fathi Milhem, Basel Musmar, Nimer Adeeb, Motaz Daraghma, Kareem El Naamani, Nils Henninger, Sri Hari Sundararajan, Anna Luisa Kühn, Jane Khalife, Sherief Ghozy, Luca Scarcia, Benjamin YQ Tan, Robert W Regenhardt, Jeremy J Heit, Nicole M Cancelliere, Joshua D Bernstock, Aymeric Rouchaud, Jens Fiehler, Sunil Sheth, Ajit S Puri, Christian Dyzmann, Marco Colasurdo, Leonardo Renieri, João Pedro Filipe, Pablo Harker, Răzvan Alexandru Radu, Mohamad Abdalkader, Piers Klein, Thomas R Marotta, Julian Spears, Takahiro Ota, Ashkan Mowla, Pascal Jabbour, Arundhati Biswas, Frédéric Clarençon, James E Siegler, Thanh N Nguyen, Ricardo Varela, Amanda Baker, Muhammed Amir Essibayi, David Altschul, Nestor R Gonzalez, Markus A Möhlenbruch, Vincent Costalat, Benjamin Gory, Christian Paul Stracke, Constantin Hecker, Hamza Shaikh, Christoph J Griessenauer, David S Liebeskind, Alessandro Pedicelli, Andrea M Alexandre, Illario Tancredi, Tobias D Faizy, Erwah Kalsoum, Max Wintermark, Boris Lubicz, Aman B Patel, Vitor Mendes Pereira, Adrien Guenego and Adam A Dmytriw in International Journal of Stroke

sj-docx-3-wso-10.1177_17474930251317883 – Supplemental material for Efficacy and safety of mechanical thrombectomy in distal medium middle cerebral artery occlusion ischemic stroke patients on low-dose aspirin Supplemental material, sj-docx-3-wso-10.1177_17474930251317883 for Efficacy and safety of mechanical thrombectomy in distal medium middle cerebral artery occlusion ischemic stroke patients on low-dose aspirin by Hamza Adel Salim, Vivek Yedavalli, Fathi Milhem, Basel Musmar, Nimer Adeeb, Motaz Daraghma, Kareem El Naamani, Nils Henninger, Sri Hari Sundararajan, Anna Luisa Kühn, Jane Khalife, Sherief Ghozy, Luca Scarcia, Benjamin YQ Tan, Robert W Regenhardt, Jeremy J Heit, Nicole M Cancelliere, Joshua D Bernstock, Aymeric Rouchaud, Jens Fiehler, Sunil Sheth, Ajit S Puri, Christian Dyzmann, Marco Colasurdo, Leonardo Renieri, João Pedro Filipe, Pablo Harker, Răzvan Alexandru Radu, Mohamad Abdalkader, Piers Klein, Thomas R Marotta, Julian Spears, Takahiro Ota, Ashkan Mowla, Pascal Jabbour, Arundhati Biswas, Frédéric Clarençon, James E Siegler, Thanh N Nguyen, Ricardo Varela, Amanda Baker, Muhammed Amir Essibayi, David Altschul, Nestor R Gonzalez, Markus A Möhlenbruch, Vincent Costalat, Benjamin Gory, Christian Paul Stracke, Constantin Hecker, Hamza Shaikh, Christoph J Griessenauer, David S Liebeskind, Alessandro Pedicelli, Andrea M Alexandre, Illario Tancredi, Tobias D Faizy, Erwah Kalsoum, Max Wintermark, Boris Lubicz, Aman B Patel, Vitor Mendes Pereira, Adrien Guenego and Adam A Dmytriw in International Journal of Stroke
