# Supplementary material for: Efficacy and safety of mechanical thrombectomy in distal medium middle cerebral artery occlusion ischemic stroke patients on low-dose aspirin
Source: Int J Stroke. 2025 Jan 28;20(6):669–78. doi: 10.1177/17474930251317883 (PMC12182599; doi:10.1177/17474930251317883)
Supplement: sj-docx-1-wso-10.1177_17474930251317883 – Supplemental material for Efficacy and safety of mechanical thrombectomy in distal medium middle cerebral artery occlusion ischemic stroke patients on low-dose aspirin [file sj-docx-1-wso-10.1177_17474930251317883.docx]

**Supplementary Table 1. Balance Diagnostics of Patient Characteristics After Propensity Score Weighting Between Patients With and Without Prestroke Low-Dose Aspirin Use. For each variable, the mean (mn) and standard deviation (sd) in the treatment (tx.mn, tx.sd) and control groups (ct.mn, ct.sd) are reported. Standardized effect sizes (std.eff.sz), test statistics (stat), and P values (pval) are provided to assess the balance between groups. The Kolmogorov-Smirnov statistic (ks) and its P value (ks.pval) are also included.**

| Variable | tx.mn | tx.sd | ct.mn | ct.sd | std.eff.sz | stat | pval | ks | ks.pval |
| --- | --- | --- | --- | --- | --- | --- | --- | --- | --- |
| Sex:Female | 0.534 | 0.499 | 0.533 | 0.499 | 0.001 | 0 | 0.991 | 0.001 | 0.991 |
| Sex:Male | 0.466 | 0.499 | 0.467 | 0.499 | -0.001 | *NA* | *NA* | 0.001 | *NA* |
| Age | 72.973 | 12.962 | 70.87 | 15.765 | 0.148 | 1.015 | 0.31 | 0.079 | 0.63 |
| hypercholesterolemia:0 | 0.648 | 0.478 | 0.684 | 0.465 | -0.079 | 6.287 | 0.005 | 0.037 | 0.005 |
| hypercholesterolemia:1 | 0.352 | 0.478 | 0.284 | 0.451 | 0.149 | *NA* | *NA* | 0.068 | *NA* |
| occlusion location.:Medium (M2) | 0.816 | 0.387 | 0.873 | 0.333 | -0.167 | 2.237 | 0.135 | 0.056 | 0.135 |
| occlusion location.:Distal (M3, M4) | 0.184 | 0.387 | 0.127 | 0.333 | 0.167 | *NA* | *NA* | 0.056 | *NA* |
| ASPECTS | 8.495 | 1.483 | 8.077 | 2.448 | 0.286 | -0.48 | 0.631 | 0.036 | 1 |
| hypertension:0 | 0.397 | 0.489 | 0.352 | 0.478 | 0.095 | 0.742 | 0.426 | 0.045 | 0.426 |
| hypertension:1 | 0.603 | 0.489 | 0.647 | 0.478 | -0.093 | *NA* | *NA* | 0.044 | *NA* |
| diabetes mellitus:0 | 0.757 | 0.429 | 0.794 | 0.404 | -0.092 | 0.753 | 0.423 | 0.037 | 0.423 |
| diabetes mellitus:1 | 0.243 | 0.429 | 0.205 | 0.404 | 0.094 | *NA* | *NA* | 0.038 | *NA* |
| atrial fibrillation:0 | 0.623 | 0.485 | 0.637 | 0.481 | -0.031 | 0.184 | 0.746 | 0.015 | 0.746 |
| atrial fibrillation:1 | 0.377 | 0.485 | 0.362 | 0.481 | 0.032 | *NA* | *NA* | 0.016 | *NA* |
| Anticoagulation prior to EVT:0 | 0.782 | 0.413 | 0.669 | 0.471 | 0.241 | 4.068 | 0.017 | 0.113 | 0.017 |
| Anticoagulation prior to EVT:1 | 0.196 | 0.397 | 0.257 | 0.437 | -0.14 | *NA* | *NA* | 0.061 | *NA* |
| IVT:0 | 0.531 | 0.499 | 0.53 | 0.499 | 0.003 | 1.046 | 0.329 | 0.002 | 0.329 |
| IVT:1 | 0.469 | 0.499 | 0.462 | 0.499 | 0.013 | *NA* | *NA* | 0.007 | *NA* |
| Stroke onset to puncture Time | 338.598 | 280.215 | 359.158 | 418.065 | -0.049 | -1.395 | 0.163 | 0.081 | 0.636 |
| Baseline NIHSS | 11.655 | 7.173 | 11.398 | 7.039 | 0.037 | 0.133 | 0.894 | 0.082 | 0.568 |
| mRS before stroke:0 | 0.729 | 0.445 | 0.632 | 0.482 | 0.201 | 2.357 | 0.046 | 0.097 | 0.046 |
| mRS before stroke:1 | 0.084 | 0.278 | 0.119 | 0.324 | -0.109 | *NA* | *NA* | 0.035 | *NA* |
| mRS before stroke:2 | 0.076 | 0.265 | 0.081 | 0.274 | -0.021 | *NA* | *NA* | 0.006 | *NA* |
| mRS before stroke:3 | 0.078 | 0.268 | 0.079 | 0.269 | -0.002 | *NA* | *NA* | 0.001 | *NA* |
| mRS before stroke:4 | 0.033 | 0.179 | 0.035 | 0.184 | -0.012 | *NA* | *NA* | 0.002 | *NA* |

***Abbreviations*: tx, treatment group; ct, control group; mn, mean; sd, standard deviation; std.eff.sz, standardized effect size; stat, test statistic; pval, P value; ks, Kolmogorov-Smirnov statistic; ks.pval, P value for the Kolmogorov-Smirnov test; EVT, endovascular thrombectomy; IVT, intravenous thrombolysis; NIHSS, National Institutes of Health Stroke Scale; mRS, modified Rankin Scale.**
