# Supplementary material for: Efficacy and safety of mechanical thrombectomy in distal medium middle cerebral artery occlusion ischemic stroke patients on low-dose aspirin
Source: Int J Stroke. 2025 Jan 28;20(6):669–78. doi: 10.1177/17474930251317883 (PMC12182599; doi:10.1177/17474930251317883)
Supplement: sj-docx-2-wso-10.1177_17474930251317883 – Supplemental material for Efficacy and safety of mechanical thrombectomy in distal medium middle cerebral artery occlusion ischemic stroke patients on low-dose aspirin [file sj-docx-2-wso-10.1177_17474930251317883.docx]

Supplementary Table 2. Periprocedural details

| **Variable***^1^* | **Overall** | **No Antiplatelet** | **Aspirin (75-100 mg)** | **P***^2^* |
| --- | --- | --- | --- | --- |
|  | N = 1354 | N = 1204 | N = 150 |  |
| **Given IVT, n (%)** | 624 (46) | 556 (47) | 68 (45) | 0.78 |
| **First line technique, n (%)** |  |  |  | 0.002 |
| ***Aspiration*** | 241 (19) | 207 (18) | 34 (24) |  |
| ***Both*** | 869 (67) | 793 (68) | 76 (54) |  |
| ***Stent retriever*** | 190 (15) | 159 (14) | 31 (22) |  |
| **Side, n (%)** |  |  |  | 0.73 |
| ***Right*** | 635 (47) | 562 (47) | 73 (49) |  |
| ***Left*** | 707 (53) | 630 (53) | 77 (51) |  |
| **Mothership versus Drip and Ship, n (%)** |  |  |  | 0.53 |
| ***Drip and ship*** | 583 (45) | 520 (46) | 63 (43) |  |
| ***Mothership*** | 704 (55) | 620 (54) | 84 (57) |  |
| **Onset to Arterial Puncture (min), Median (IQR)** | 266 (177, 426) | 270 (180, 442) | 230 (154, 379) | 0.007 |
| **Puncture to Recanalization Time (min), Median (IQR)** | 33 (23, 54) | 33 (23, 53) | 38 (24, 65) | 0.009 |
| **Onset to Recanalization (min), Median (IQR)** | 325 (225, 509) | 330 (231, 514) | 294 (193, 440) | 0.006 |
| **Onset to IVT needle Time (min), Median (IQR)** | 153 (100, 254) | 154 (100, 265) | 149 (104, 206) | 0.42 |
| **Pre-operative SBP, Median (IQR)** | 148 (131, 165) | 148 (131, 165) | 149 (130, 163) | 0.93 |
| **Pre-operative DBP, Median (IQR)** | 83 (74, 95) | 84 (74, 96) | 82 (73, 92) | 0.14 |
| **Pre-operative Temperature (Celsius), Median (IQR)** | 36.50 (36.20, 36.80) | 36.50 (36.20, 36.90) | 36.60 (36.30, 36.80) | 0.31 |
| **Anesthesia, n (%)** |  |  |  | <0.001 |
| ***CS/LA*** | 904 (70) | 849 (74) | 55 (42) |  |
| ***GA*** | 379 (30) | 303 (26) | 76 (58) |  |
| **Puncture site, n (%)** |  |  |  | 0.4 |
| ***Femoral*** | 823 (97) | 684 (97) | 139 (99) |  |
| ***Radial*** | 23 (2.7) | 21 (3.0) | 2 (1.4) |  |
| **Imaging after MT, n (%)** |  |  |  | <0.001 |
| ***CT*** | 875 (68) | 809 (71) | 66 (46) |  |
| ***Both*** | 191 (15) | 159 (14) | 32 (23) |  |
| ***MRI*** | 217 (17) | 173 (15) | 44 (31) |  |
| ***No Imaging*** | 6 (0.5) | 6 (0.5) | 0 (0) |  |
| **Total Number of Passes, Median (IQR)** | 2.00 (1.00, 3.00) | 2.00 (1.00, 3.00) | 1.00 (1.00, 2.00) | 0.2 |
| **Embolization in new territories, n (%)** | 43 (3.3) | 38 (3.3) | 5 (3.8) | 0.8 |
| **Perforation, n (%)** | 19 (1.5) | 18 (1.6) | 1 (0.8) | 0.71 |
| **Artery Dissection, n (%)** | 12 (0.9) | 12 (1.0) | 0 (0) | 0.62 |
| *^1^* Abbreviations: IVT = Intravenous Thrombolysis , SBP = Systolic Blood Pressure , DBP = Diastolic Blood Pressure, CS/LA = Conscious Sedation/Local Anesthesia , GA = General Anesthesia | | | | |
| *^2^* Pearson’s Chi-squared test; Wilcoxon rank sum test; Fisher’s exact test | | | | |
