# Supplementary material for: Efficacy and safety of mechanical thrombectomy in distal medium middle cerebral artery occlusion ischemic stroke patients on low-dose aspirin
Source: Int J Stroke. 2025 Jan 28;20(6):669–78. doi: 10.1177/17474930251317883 (PMC12182599; doi:10.1177/17474930251317883)
Supplement: sj-docx-3-wso-10.1177_17474930251317883 – Supplemental material for Efficacy and safety of mechanical thrombectomy in distal medium middle cerebral artery occlusion ischemic stroke patients on low-dose aspirin [file sj-docx-3-wso-10.1177_17474930251317883.docx]

Supplementary Table.3: Univariable Crude Analysis and doubly robust IPTW Evaluation of Outcomes Aspirin (Versus No Antiplatelet)

| **Variable***^1^* | **No Antiplatelet** | **Aspirin (75-100 mg)** | *Crude Analysis* | | *Doubly Robus IPTW model* | |
| --- | --- | --- | --- | --- | --- | --- |
|  | N = 1204 | N = 150 | **OR** **(95% CI)***^1^* | **p-value** | **OR** **(95% CI)***^1^* | **p-value** |
| *Day one NIHSS, Median (IQR)* | 6 (2, 14) | 4 (2, 12) | -2.2 (-3.9 to -0.55) | **0.009** | -1.5 (-2.8 to -0.27) | **0.018** |
| *NIHSS shift, Median (IQR)* | -2 (-6, 1) | -2 (-6, 1) | -0.49 (-2.0 to 1.0) | 0.52 | -1.1 (-2.4 to 0.16) | **0.086** |
| *TICI 2b-3, n (%)* | 989 (86) | 125 (88) | 1.24 (0.75 to 2.18) | 0.43 | 1.08 (0.55 to 2.09) | 0.83 |
| *TICI 2c-3, n (%)* | 618 (53) | 87 (61) | 1.38 (0.97 to 1.98) | 0.079 | 1.40 (0.90 to 2.17) | 0.14 |
| *FPE, n (%)* | 332 (31) | 54 (38) | 1.39 (0.96 to 1.99) | 0.075 | 1.20 (0.77 to 1.87) | 0.42 |
| *90-day mRS 0-1, n (%)* | 385 (40) | 67 (46) | 1.30 (0.91 to 1.84) | 0.14 | 1.62 (0.98 to 2.67) | 0.06 |
| *90-day mRS 0-2, n (%)* | 539 (56) | 94 (65) | 1.46 (1.02 to 2.12) | **0.04** | 1.89 (1.14 to 3.12) | **0.013** |
| *90-day Mortality, n (%)* | 162 (17) | 19 (13) | 0.75 (0.44 to 1.22) | 0.27 | 0.56 (0.32 to 1.00) | **0.048** |
| *sICH, n (%)* | 76 (6.6) | 10 (6.9) | 1.05 (0.50 to 1.99) | 0.89 | 0.84 (0.39 to 1.82) | 0.67 |
| *ICH (any type), n (%)* | 395 (35) | 53 (37) | 1.09 (0.76 to 1.56) | 0.64 | 0.92 (0.60 to 1.43) | 0.72 |
| *ICH (by type), n (%)* |  |  |  |  |  |  |
| *HI1* | 233 (21) | 6 (4.3) | 0.17 (0.07 to 0.35) | **<0.001** | 0.12 (0.05 to 0.31) | **<0.001** |
| *HI2* | 24 (2.2) | 3 (2.2) | 0.99 (0.23 to 2.89) | **0.99** | 1.32 (0.41 to 4.30) | **0.64** |
| *PH1* | 24 (2.2) | 8 (5.8) | 2.75 (1.14 to 5.99) | 0.016 | 1.39 (0.49 to 3.96) | 0.53 |
| *PH2* | 16 (1.5) | 14 (10) | 7.61 (3.58 to 16.0) | **<0.001** | 5.80 (2.43 to 13.8) | <0.001 |
| *SAH* | 83 (7.5) | 19 (14) | 1.95 (1.11 to 3.25) | **0.014** | 1.79 (0.93 to 3.42) | **0.08** |
| *^1^* Abbreviations: mRS = Modified Rankin Scale , NIHSS = National Institutes of Health Stroke Scale, sICH= symptomatic intracerebral hemorrhage , ICH= intracerebral hemorrhage | | | | | | |
| *^2^* OR = Odds Ratio, CI = Confidence Interval/ Beta was only estimated for Day one NIHSS and NIHSS shift | | | | | | |
